# Head and neck NUT carcinoma—radiotherapy dose, chemotherapy choice, and outcomes: lessons from a 7-year complete remission

**DOI:** 10.1007/s00066-026-02552-x

**Published:** 2026-06-01

**Authors:** Lara Schulz, Matthias Zähringer, Klaus-Henning Kahl, Nikolaos Balagiannis, Bertram Jehs, Johannes Doescher, Johannes Zenk, Georg Stüben, Maria Neu

**Affiliations:** 1 Department of Radiotherapy and Radiation Oncology, Faculty of Medicine, University of Augsburg, Stenglinstr. 2, 86156 Augsburg, Germany; 2 Comprehensive Cancer Center Augsburg (CCCA), Faculty of Medicine, University of Augsburg, 86156 Augsburg, Germany; 3 Comprehensive Cancer Center Alliance WERA (CCC WERA), 86156 Augsburg, Germany; 4 Bavarian Cancer Research Center (BZKF), 86156 Augsburg, Germany; 5 Department of Diagnostic and Interventional Radiology and Neuroradiology, Faculty of Medicine, University of Augsburg, 86156 Augsburg, Germany; 6 Department of Otorhinolaryngology and Head & Neck Surgery, University Hospital Augsburg, 86156 Augsburg, Germany

**Keywords:** NUTM1 gene rearrangement, Supraglottic laryngeal cancer, Accelerated fractionation, Long-term survival, Combined modality therapy

## Abstract

**Background:**

NUT carcinoma (NUTc) is a rare, aggressive squamous carcinoma defined by *NUTM1* rearrangements. Outcomes in head and neck (H&N) disease are poor, and no uniform treatment standard exists; available evidence largely derives from case reports and small series.

**Methods:**

We performed a structured narrative review of H&N NUTc with an emphasis on radiotherapy (RT) dose concepts, chemotherapy regimens, and outcome signals.

**Results:**

Published evidence indicates poor overall survival, particularly in metastatic disease. Long-term remissions have been described in selected patients with non-metastatic disease treated with multimodal approaches combining surgery, multi-agent chemotherapy, and early integrated RT. Across reports, use of RT in first-line management and delivery of definitive-dose RT are repeatedly associated with better outcomes. Evidence for immune checkpoint inhibitors is limited to anecdotal single-patient reports.

**Case vignette:**

A 19-year-old man with locally advanced supraglottic NUTc and bilateral cervical nodal disease (M0) presented with dysphagia and otalgia. Following laser debulking and temporary tracheostomy, induction cisplatin/doxorubicin/ifosfamide led to a complete metabolic response in positron-emission tomography/computed tomography (PET/CT) after two cycles of chemotherapy. Definitive PET/CT-guided intensity-modulated (IM)RT/image-guided (IG)RT was delivered to 73.2 Gy using an accelerated twice-daily schedule (2.0 Gy to the planning target volume [PTV] in the morning and 1.6 Gy boost to the initial gross target volume [GTV] in the afternoon; ≥ 8-hour interval). Acute toxicity was grade 3 dermatitis and grade 2 mucositis (CTCAE v5.0). After RT, chemotherapy was continued as consolidation and completed with vincristine/doxorubicin/ifosfamide (six cycles) due to acute kidney injury. Complete remission is ongoing at > 7 years, with persistent xerostomia and dysgeusia.

**Conclusion:**

In selected non-metastatic H&N NUTc, durable control has been reported with timely systemic therapy plus curative-intent RT, often exceeding 50 Gy. This review summarizes dose concepts and case-based systemic regimens and provides a comparative table to support individualized treatment planning.

## Introduction

NUT carcinoma (NUTc) is an aggressive squamous carcinoma defined by rearrangements of the *NUTM1* gene (15q14), most commonly *BRD4–NUTM1*. Tumors typically show diffuse, speckled nuclear NUT staining on immunohistochemistry using the C52 antibody, with confirmatory testing by fluorescence in situ hybridization (FISH) and/or sequencing where available [1, 2]. Primary sites include the midline thorax and the head and neck (H&N), and many patients present with advanced disease; outcomes remain poor despite multimodal therapy [1, 3–5]. Most cases harbor *BRD4–NUTM1 *fusions, whereas the remainder show variant *NUTM1 *rearrangements including *BRD3* and other partners [2]. Because NUT carcinoma is newly recognized and often misdiagnosed, its true incidence remains unknown [6].

For H&N disease, timely diagnostic confirmation and early multidisciplinary planning are therefore essential, ideally including molecular characterization of the fusion partner when feasible [7]. Treatment decisions are frequently made in the absence of a uniform standard, local therapy is often challenged by rapid progression, and distant failure is common, highlighting the need for effective local and systemic strategies [3–5]. While durable remissions have been described in selected patients with non-metastatic disease, published evidence remains heterogeneous and largely case based.

Here, we provide a structured narrative review of H&N NUTc with an emphasis on radiotherapy (RT) dose concepts, systemic regimens used alongside RT, and recurring prognostic signals. We additionally present a brief clinical vignette illustrating a dose-intensive, image-guided RT approach with long-term disease control beyond 7 years.

## Methods

We conducted a structured narrative review of head and neck (H&N) NUT carcinoma. We searched PubMed/MEDLINE and Embase from database inception to December 2025 using free-text keywords and controlled vocabulary (MeSH/Emtree) related to NUT carcinoma and H&N management (“NUT carcinoma,” “NUT midline carcinoma,” “NUTM1,” “head and neck,” “larynx,” “parotid,” “sinonasal,” “radiotherapy,” “chemoradiotherapy,” “chemotherapy”).

We included reviews, registry analyses, case series, and case reports that described H&N presentations or provided information applicable to H&N radiotherapy dose, technique, systemic regimens used alongside RT, and outcomes with extractable treatment details. We excluded duplicates and conference abstracts without extractable data. Reference lists of eligible articles were also screened. Non-English full texts were excluded when reliable translation was not available.

Two authors independently screened records and extracted data; discrepancies were resolved by consensus. Extracted variables included anatomic site and stage, surgical approach (if applicable), radiotherapy dose/fractionation and technique, timing of systemic therapy relative to RT (concurrent/interdigitated vs. sequential), and outcome/follow-up. Due to heterogeneity and the predominance of case-level evidence, results were synthesized qualitatively without meta-analysis; regimen and sequencing statements are presented as descriptive, case-based observations rather than recommended standards.

## Results

### Prognosis and stage

In an individual patient data (IPD) analysis (*n* = 119), median overall survival (OS) was 5 months; 1‑ and 5‑year OS were approximately 25% and 7%, respectively [4]. Outcomes differed markedly by baseline stage: patients with non-metastatic disease had 1‑/2-/5-year OS of 31.7%/8.7%/8.7% versus 9.8%/0%/0% for metastatic disease (*p* = 0.004) [4]. In a dedicated H&N cohort, median OS and progression-free survival (PFS) were 9.7 and 6.6 months, respectively, with a 2-year OS of 30% [3].

### Radiotherapy dose and technique

Across published H&N reports, definitive-dose RT doses most commonly range from 50 to 70 Gy, whereas postoperative RT spans a broader range (24–66 Gy) [4, 7]. In the IPD analysis, inclusion of RT in initial management was associated with improved survival (1-year 37.7% vs. 13%; *p* = 0.001), and doses > 50 Gy were associated with better OS (*p* = 0.0038) [4]. Contemporary H&N reports predominantly employ intensity-modulated radiotherapy (IMRT), frequently with image guidance [7–9]. Illustrative cases include organ-preserving laryngeal IMRT (60 Gy in 30 fractions to the high-risk volume and 54 Gy in 30 fractions to the elective volume) with remission beyond 2 years [9] and a skull-base/neck presentation treated with 50 Gy in 20 fractions to the primary and bilateral neck with concurrent docetaxel/cisplatin, followed by early distant progression [10].

### Systemic therapy used with RT

No uniform systemic regimen exists; most published data are case based [4, 5, 11]. In the IPD analysis, inclusion of chemotherapy in first-line treatment was associated with improved overall survival, with site-specific significance in mediastinal primaries [4]. Regimens reported with definitive RT up to 70 Gy include concurrent cisplatin (80 mg/m^2^ every 3 weeks) in supraglottic disease, achieving local complete response but followed by early distant progression [12]. For regimen context from non-H&N cases, durable complete remissions have been described with 60 Gy delivered concurrently with ifosfamide/etoposide (often with additional IE/VAC cycles) or with weekly carboplatin/paclitaxel, maintained for 18–21 months [11]. In a pediatric two-patient series, chemotherapy followed the Scandinavian Sarcoma Group protocol SSG IX, alternating VAI (vincristine/doxorubicin/ifosfamide) and PAI (cisplatin/doxorubicin/ifosfamide). RT was integrated early after limited surgery with curative intent, delivering 54 Gy in one patient (completed with conventional fractionation after an intended hyperfractionated start) and IMRT 59.4 Gy/33 fractions to the primary/involved nodes with 50.4 Gy to the contralateral neck in the other. It was initiated at the end of cycle 1, and chemotherapy was restricted to three cycles, with ^18^F-fluorodeoxyglucose (FDG)-PET complete response already documented during cycle 1 in both patients [8].

### Surgery and organ preservation

When microscopically margin-negative resection (R0) is feasible, improved outcomes have been reported in H&N cohorts [3]. Organ-preserving approaches have also been described, including transoral CO_2_ excision followed by IMRT in anatomically limited laryngeal disease [9]. For locally advanced laryngeal/supraglottic presentations, definitive chemoradiation remains a common curative-intent strategy in published cohorts [3, 4].

### Toxicity and follow-up

Acute mucositis and dermatitis are frequently reported and typically graded according to Common Terminology Criteria for Adverse Events (CTCAE) v5.0 [13]. Early failures are frequently reported, often within the first year, supporting early post-treatment imaging and close surveillance, particularly in the first 2 years [4, 10, 12].

## Case vignette

### Presentation and diagnostic work-up

**Fig. 1:**
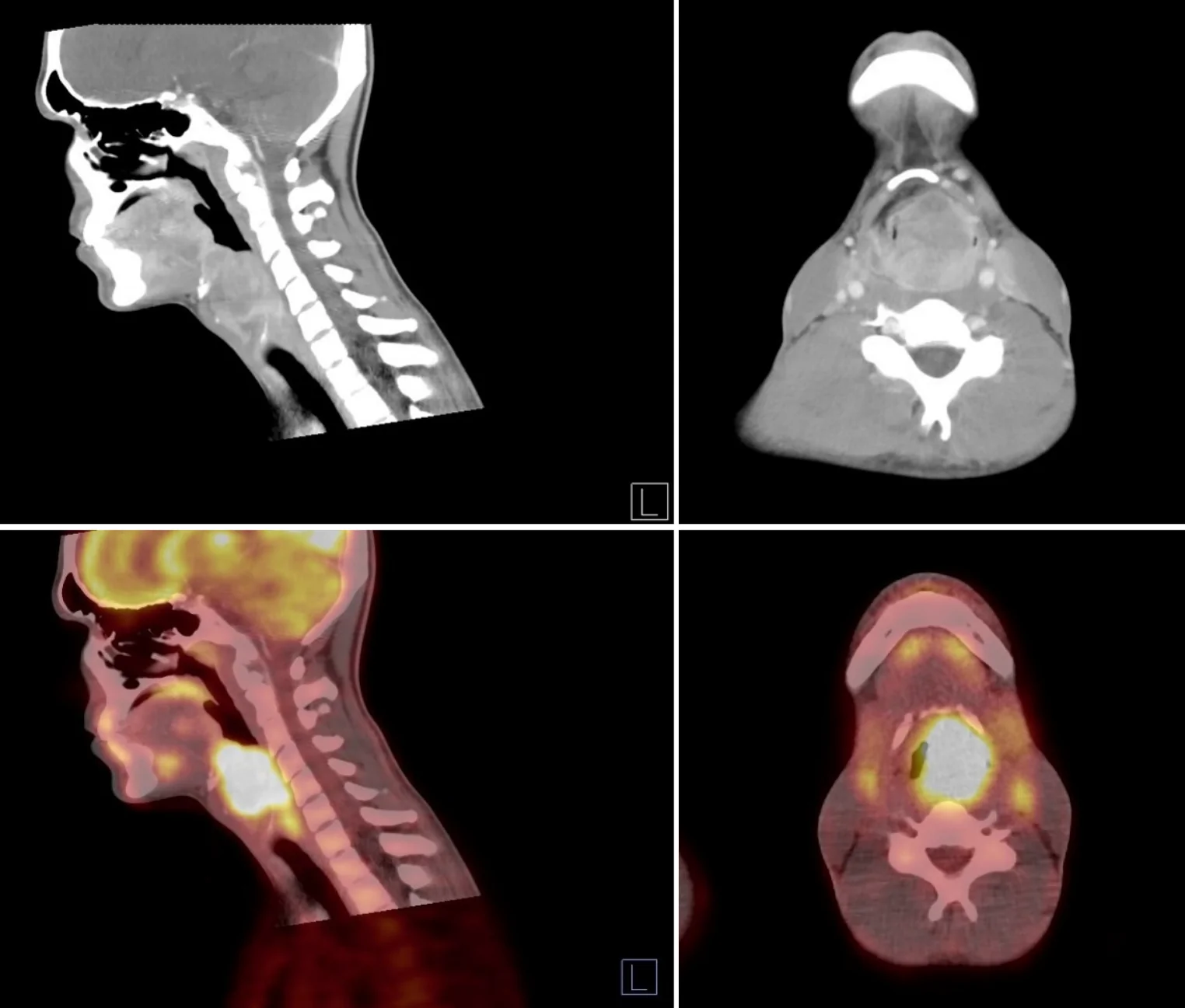
Baseline imaging. Contrast-enhanced computed tomography (CT) demonstrating a left supraglottic mass crossing the midline with extension to the hypopharynx and tongue base. ^18^F-fluorodeoxyglucose (FDG) positron emission tomography/CT (PET/CT) (fused) showing an FDG-avid supraglottic primary with bilateral cervical nodal uptake

A 19-year-old man with no relevant medical history and no smoking history presented with 6 weeks of progressive dysphagia and left-sided otalgia. Panendoscopy revealed a bulky left supraglottic mass. Initial histology showed small round blue cells with a Ki-67 index close to 100%, and the initial diagnosis remained uncertain. Baseline PET/CT demonstrated a 3.8 × 4.2 × 4.9 cm FDG-avid supraglottic tumor crossing the midline with extension to the hypopharynx and tongue base, as well as bilateral FDG-avid cervical lymph nodes along the carotid sheath up to the retromandibular region; no distant metastases were detected (Fig. 1). Following laser debulking, a temporary tracheostomy was required due to postoperative edema and bleeding. Immunohistochemistry at a reference laboratory confirmed NUT carcinoma; fusion partner testing was not performed because it was not routinely available at that time.

### Systemic therapy and early response

Induction chemotherapy with cisplatin/doxorubicin/ifosfamide (every 3 weeks) resulted in a complete metabolic response on PET/CT after two cycles.

### Radiotherapy planning and delivery

**Fig. 2:**
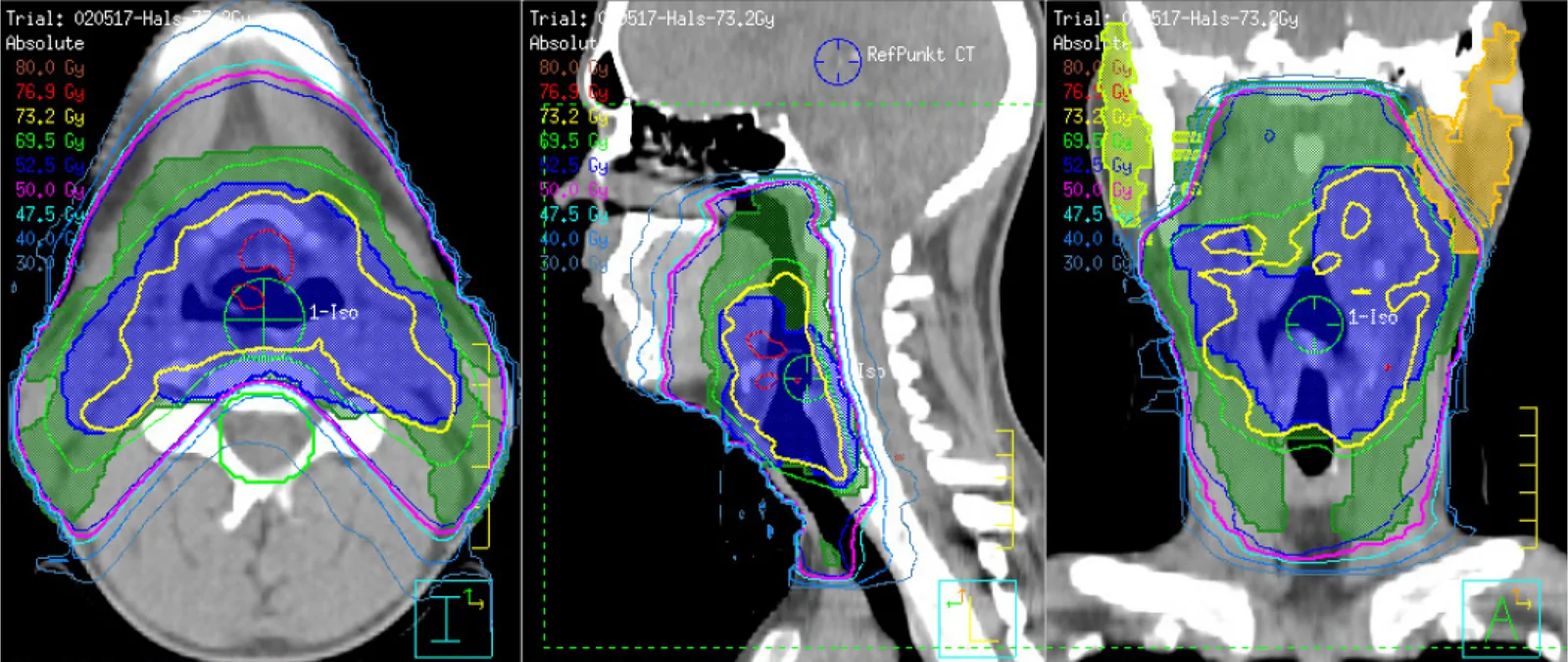
Intensity-modulated radiotherapy (IMRT) treatment plan: PET/CT-based target volumes with cumulative 73.2 Gy to initial gross disease (primary and nodes) and 54 Gy to elective regions

Definitive RT was administered using PET/CT-guided IMRT/IGRT. Phase 1 delivered 30 Gy in 15 fractions to the supraglottic/hypopharyngeal region (including the tongue base) and regional lymphatics. Phase 2 consisted of an accelerated twice-daily boost over 12 treatment days: 2.0 Gy in the morning to the high-risk volume and 1.6 Gy in the afternoon (≥ 8-hour interval) to the initial gross disease (primary and involved nodes). The cumulative dose was 73.2 Gy to initial gross disease and 54 Gy electively. Dose escalation was used to intensify coverage of the initial gross disease while maintaining elective nodal treatment; organ-at-risk constraints followed institutional head-and-neck IMRT practice. Dose distribution and isodose levels for the RT plan are provided in Fig. 2.

### Toxicity, consolidation, and outcome

**Fig. 3:**
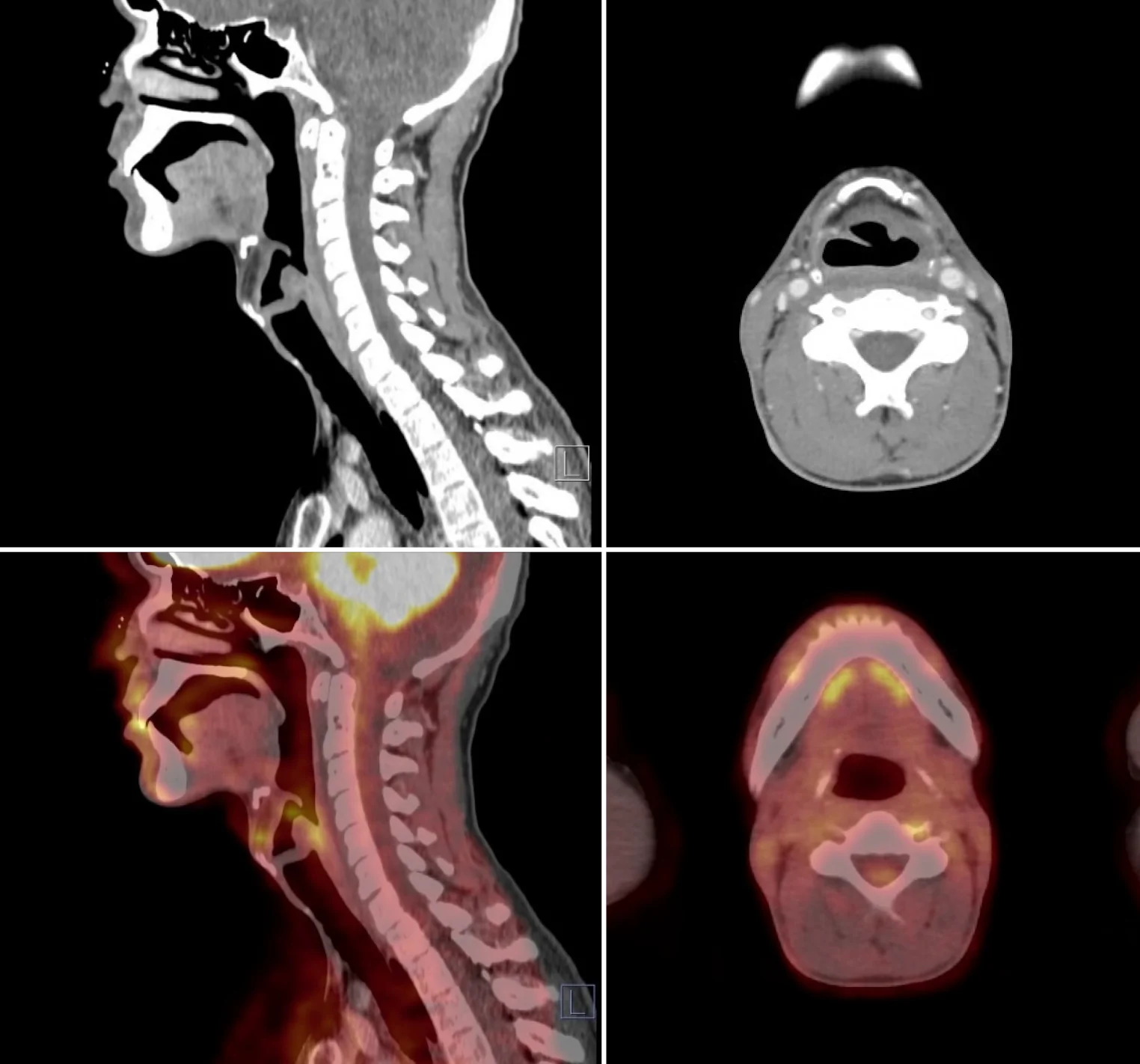
Five-year follow-up imaging after treatment. Contrast-enhanced CT demonstrates marked regression with no residual measurable disease. Corresponding fused FDG PET/CT shows complete metabolic response at the primary site and cervical lymph nodes

Acute toxicity included CTCAE v5.0 grade 3 dermatitis and grade 2 mucositis, with edema causing dysphagia and mild dyspnea, managed with a short course of corticosteroids, gastric protection, and analgesics [13]. Chemotherapy was resumed 5 weeks after RT and completed with vincristine/doxorubicin/ifosfamide for six cycles after acute kidney injury. The patient remains in complete remission more than 7 years after treatment, with persistent xerostomia and dysgeusia and preserved laryngeal function. Representative follow-up imaging at 5 years confirmed complete metabolic response (Fig. 3); ongoing remission was confirmed by serial clinical assessments and imaging, including PET/CT, without evidence of local or distant relapse at last follow-up.

### How the vignette fits

This vignette illustrates dose-intensive, image-guided RT (73.2 Gy to initial gross disease) combined with an Ewing-like multi-agent chemotherapy sequence in a non-metastatic setting, aligning with the recurring patterns identified in published H&N NUTc experience [3, 4, 9, 11, 14]. To place this case in context and to summarize comparable dose–regimen–outcome profiles reported in the literature, Table 1 provides a focused cross-case overview.

**Table 1:** Selected H&N NUTc cases with RT dose and outcome (non-H&N cases included for regimen context)

Author (year)	Site/stage	Age	Treatment summary	Regimen (ref.)	Radiotherapy (RT)	Outcome/follow-up
This report	Supraglottic + bilateral cervical nodes, M0	19	Laser debulking → induction chemo (PET-CR after 2 cycles) → PET-based IMRT/IGRT (accelerated) → consolidation chemo	Ewing-like sequence: cisplatin/doxorubicin/ifosfamide (induction) → vincristine/doxorubicin/ifosfamide (consolidation); *sequential* (no concurrent chemo)	73.2 Gy to initial gross disease (accelerated BID: 2.0 Gy AM + 1.6 Gy PM boost to GTV), 54 Gy elective	CR; disease free at 7+ years
Storck et al. (2017) [8]—Case 1	Sublingual gland primary; cervical nodes; progression during diagnostic work-up to right supraclavicular + left mediastinal node	9	Submandibulectomy + partial neck dissection → early RT to all involved sites starting end of cycle 1	SSG IX VAI/PAI; RT started end of cycle 1; *interdigitated*; chemo limited to 3 cycles	Intended 1.5 Gy BID; stopped after 30 Gy (mask-related pain) → continued under anesthesia with 2.0 Gy QD to total 54 Gy (all involved regions treated simultaneously)	FDG-PET CR during cycle 1 prior to RT; CR > 6 years; late effect: soft tissue hypoplasia in irradiated area
Storck et al. (2017) [8]—Case 2	Masseter/parotid-adjacent primary; cervical nodal disease (levels I–II; multiple nodes); positive margins; LVI	9	Debulking (~80%) + cervical node surgery → early IMRT starting end of cycle 1 (includes contralateral neck)	SSG IX VAI/PAI; 3/4 planned cycles; RT started end of cycle 1; *interdigitated*	IMRT 59.4 Gy/33 (1.8 Gy QD) to primary + initially involved nodes; 50.4 Gy to contralateral neck	FDG-PET CR during cycle 1 prior to RT (post-op changes noted); no progression 8 months post-treatment; asymptomatic EF decline (0.63 → 0.48)
Zhang et al. (2020) [9]	Larynx, limited stage; R0 after CO_2_ laser	20	Local CO_2_ laser excision (R0) → definitive IMRT	–	60 Gy/30 fx (high-risk); 54 Gy/30 fx (elective)	Continuous remission ~26 months
Higashino (2022) [12]	Supraglottis, cT3N2bM0 (H&N)	22	Definitive RT + cisplatin (concurrent)	Cisplatin 80 mg/m^2^ q3w; *concurrent*	70 Gy/35 (2 Gy × 35)	CR locally; ovarian/peritoneal metastases; death ~32 weeks
Zhao 2024 [14]	Parotid, residual post-op disease	52	Post-op systemic therapy; planned adjuvant RT stopped early	Paclitaxel/cisplatin → nab-paclitaxel/cetuximab/cisplatin; peri-RT intent; *RT incomplete*	66 Gy/30 fx planned; RT stopped after 12 fx at 26.4 Gy	OS 7 months
Elkhatib (2019) [10]	Skull base/sella, cervical nodes (H&N)	47	Transsphenoidal debulking → skull-base RT → bilateral neck RT with concurrent chemo	Docetaxel/cisplatin; *concurrent with bilateral neck RT* (after skull-base RT)	50 Gy/20 fx (skull base); 50 Gy/20 fx (bilateral neck)	Early distant progression; OS 5 months
Pan et al. (2020) [11]—Case 1	Mediastinum/neck, unresectable	31	Definitive RT with concurrent chemo; additional cycles after RT	Ifosfamide/etoposide (IE) *concurrent*; further IE/VAC afterwards	60 Gy/30 fx EBRT (definitive)	CR soon after CRT; ongoing 18 months
Pan et al. (2020) [11]—Case 2	Mediastinum, unresectable	70	Definitive RT with concurrent weekly chemo (one week skipped)	Weekly carboplatin/paclitaxel; *concurrent*	60 Gy/30 fx EBRT (10 Gy boost within the 60 Gy)	CR ~13 months after start; maintained 21 months

*BID* twice daily, *CR* complete remission, *CRT* chemoradiotherapy, *EBRT* external-beam radiotherapy, *EF* ejection fraction, *FDG-PET* fluorodeoxyglucose positron-emission tomography, *fx* fractions, *GTV* gross tumor volume, *H&N* head and neck, *IGRT/IMRT* image-guided/intensity-modulated radiotherapy, *LVI* lymphovascular invasion, *ND* neck dissection, *OS* overall survival, *PAI* cisplatin/doxorubicin/ifosfamide, *q3w* every 3 weeks, *QD* once daily, *R0* microscopically margin-negative resection, *SSG* Scandinavian Sarcoma Group, *VAI* vincristine/doxorubicin/ifosfamide

## Discussion

This synthesis supports three consistent clinical signals. First, baseline stage is a dominant prognostic factor: non-metastatic disease shows substantially better survival than metastatic presentations in pooled analyses, and early complete response appears to track with improved outcomes in H&N cohorts [3, 4].

Second, local therapy is central in curative-intent management. When negative-margin resection is feasible, cohort data suggest improved outcomes with surgery followed by adjuvant RT/chemoradiotherapy (CRT) [3]; in anatomically limited laryngeal disease, organ-preserving approaches combining local excision and IMRT have achieved durable control in individual reports [9]. Across published experience, incorporation of radiotherapy in first-line care and delivery of definitive-dose treatment (often > 50 Gy) are recurring features in patients with more favorable outcomes, although early distant failure remains common despite good local response [4, 12]. Where feasible, treatment acceleration (e.g., hyperfractionation or other overall treatment time–shortening approaches) may be worth considering in selected curative-intent cases. This is supported by recurring case-level patterns—including our case and pediatric experience in which RT was initiated early and an accelerated start was attempted—suggesting that timely, definitive local therapy may matter in this aggressive disease; however, evidence remains limited and practice should remain individualized [8].

Third, systemic therapy appears important: complete remissions have been reported with concurrent chemoradiation using ifosfamide/etoposide or weekly carboplatin/paclitaxel, yet comparative evidence is lacking and these schedules should not be interpreted as standards [11]. Where multi-agent chemotherapy is pursued with curative intent, protocols aligned with Ewing sarcoma regimens (e.g., VAC/IE- or VAI/PAI-like approaches) represent a biologically plausible template that has been used in our and pediatric experience, but this signal remains hypothesis generating and requires validation [8].

### Follow-up and surveillance

Early distant failure is frequently reported, often within the first year. Early post-treatment imaging and close, short-interval surveillance during the first 2 years may therefore be reasonable, particularly after chemoradiotherapy [4, 10, 12].

### Emerging systemic options and trials

Evidence for PD-1–based approaches in NUT carcinoma remains limited to case reports and cannot define standard practice [15, 16]. Given the mechanistic relevance of BET proteins, BET-targeted strategies are being evaluated in early-phase trials, including ZEN003694-based combinations (NCT05019716; NCT05372640) and a dual BET/CBP–p300 inhibitor (NCT05488548); clinical efficacy and optimal sequencing with curative-intent RT remain to be established [17].

### Limitations and research needs

The evidence base is dominated by case reports and small series and is prone to selection and reporting bias. Regimen and dose details are variably reported, and fusion partner status is inconsistently documented, limiting inference on prognostic or predictive subgroups [3, 4, 14]. Prospective registries and multi-institutional datasets are needed to standardize reporting (dose, technique, sequencing), define systemic partners for RT, and refine patient selection. Fusion heterogeneity may contribute to clinical variability; routine reporting of *NUTM1* fusion partners should be encouraged, as supported by recent reports of uncommon fusions with atypical courses [18].

## Conclusion

For non-metastatic H&N NUT carcinoma, durable control has been reported with multimodal, curative-intent strategies that combine timely systemic therapy and definitive-dose radiotherapy, often exceeding 50 Gy. Even with strong local responses, early distant failure remains a major risk, supporting close early surveillance. Given the predominance of case-level evidence, systemic regimens and sequencing signals should be considered hypothesis generating; prospective registries and trial enrollment, including BET-targeted studies, are key priorities to refine standards and patient selection [3, 4, 11, 12, 17].

## Data Availability

This is a narrative review with an illustrative, de-identified patient vignette. All data supporting the review are contained within the article and its tables/figures. De-identified source data from the vignette (e.g., imaging excerpts and RT plan screenshots) can be shared by the corresponding author upon reasonable request, subject to institutional policy and patient privacy.
